# The Coincidence of Newly Diagnosed Type 1 Diabetes Mellitus with IgM Antibody Positivity to Enteroviruses and Respiratory Tract Viruses

**DOI:** 10.1155/2018/8475341

**Published:** 2018-08-16

**Authors:** Murat Karaoglan, Fahriye Eksi

**Affiliations:** ^1^Division of Pediatric Endocrinology, Gaziantep University Faculty of Medicine, Gaziantep, Turkey; ^2^Department of Medical Microbiology, Gaziantep University Faculty of Medicine, Gaziantep, Turkey

## Abstract

**Objective:**

Viruses trigger and promote islet cell destruction and cause type 1 diabetes mellitus (T1DM). However, the existence of a cause-and-effect relationship is under debate. The aim of this study is to investigate the sero-epidemiological and molecular evidence on enteroviruses and respiratory viruses in patients with newly diagnosed T1DM during the cold season.

**Design:**

Forty children newly diagnosed with T1DM and 30 healthy children who presented to the clinic over the course of a year were included in the study. The IgM antibodies against enteroviruses and respiratory viruses were studied using the indirect immunofluorescence assay (IFA) test, and no CBV4-specific RNA was detected in the children. The onset times of T1DM were classified into fall-winter and spring-summer seasons and separated into cold, moderate, or warm months in terms of temperature.

**Results:**

The percentages of viral IgM antibodies against most common viruses were detected in the patients as follows: influenza B (IVB) (70%), echovirus 7 (ECHO7) (45%), parainfluenza virus 4 (PIV4) (40%), coxsackievirus A7 (CAV7) (27.5%), and H3N2 (22.5%). Compared with the control group, the above viruses had a significant association with T1DM (*p* ≤ 0.001, *p* ≤ 0.001, *p* = 0.035, *p* = 0.003, and *p* = 0.023, resp.). CBV4-specific RNA was not detected in any serum. A total of 75% and 95% patients were diagnosed with T1DM in the fall-winter seasons and cold-moderate months, respectively.

**Conclusion:**

Our study demonstrates the significant association between T1DM and the presence of IgM antibodies against IVB, ECHO7, PIV4, CAV7, and H3N2, and the majority of newly diagnosed T1DM appeared in the fall-winter season. It suggests that enteroviruses and respiratory viruses, in addition to seasonal variation, could play a role in the etiopathogenesis and clinical onset of T1DM.

## 1. Introduction

Many studies in recent decades have determined that enteroviruses (EV), in particular, and respiratory viruses play a role in the pathogenesis of type 1 diabetes (T1DM) [1–3]. Respiratory viruses share common clinical and pathological characteristics with enteroviruses. These viruses can grow in either the respiratory or intestinal tract. Most enteroviruses exhibit tropism to islet cells. In experimental and epidemiologic studies, it has been shown that influenza viruses can affect islet cells [4, 5]. Moreover, viruses lead to beta cell destruction through indirect or direct pathways [6, 7]. These patterns are known as T1a (autoimmune) and T1b (nonimmune or cytopathic).

The onset time of T1DM exhibits seasonal variations [8]. The seasonal pattern coincides with the typical influenza season seen during the fall and winter season [4, 9]. In addition, the incidence of T1DM has been demonstrated to increase during the aftermath of outbreaks of influenza and mumps [10]. Likewise, many viruses, including respiratory viruses and enteroviruses, lead to beta cell destruction after recurrent, cumulative, and prolonged chronic inflammation with a “multiple hit” of viruses. The onset of T1DM appears by means of a “final hit” of viruses after a long unapparent process [11]. Moreover, mixed viruses trigger and potentiate each other during the process of target tissue damage [12].

T1DM is one of the most chronic and dysmetabolic childhood disorders, resulting from an interaction between the host immune system and heterogeneous environmental factors in the polygenic background [7]. Numerous longitudinal, epidemiological, animal, and human modeling studies have been conducted using sero-epidemiological, molecular, and pathological evidence, indicating that viruses are strongly linked with T1DM [6, 7].

Gut microbiota is actively involved in the interplay between the host immune system and islet destruction [13]. In support of this idea, many studies have been documented in recent decades that have related the alteration in gut microbiota to a pathological process, leading to autoimmune diabetes [14]. In addition, a prospective study showed that a sibling of a child newly diagnosed with T1DM who was EV-seropositive developed T1DM after intrafamilial transmission of EV [15].

The present study, the first to include seasonal variations in Turkey, has been designed to investigate the association between T1DM and multiple EV and respiratory tract viruses, which are postulated to be diabetogenic viruses.

## 2. Materials and Methods

The study was carried out retrospectively. The medical records of children who were newly diagnosed with T1DM between September 2013 and October 2014 in Gaziantep Province, Southeast Turkey, were collected. We included 40 children aged 1–16 years (mean age = ±3.85 years) who were newly diagnosed with T1DM and 30 healthy children who had presented to pediatric policlinics during the same period. The one-year period was classified into two groups to determine time variations in the spread of a virus: group 1 = fall/winter (September to February) and spring/summer (March to August) and group 2 = cold months (November, December, January, and February), moderate months (September, October, March, and April), and warm months (May, June, July, and August). In respect of temperature and climate, seasons and months were classified according to the Köppen-Geiger climate classification for Gaziantep Province [16]. The DMS latitude and longitude coordinates for Gaziantep are 37°03′33.98^″^N and 37°22′57.00^″^E, respectively. Type C climate is dominant in Gaziantep.

T1DM was diagnosed using the following criteria: history of polyuria and/or polydipsia, blood glucose above 200 mg/dL, low insulin and C-peptide level, glycated hemoglobin A1C (HbA1C) above 6.5%, and insulin dependency. Blood was drawn from patients with newly diagnosed T1DM for the following assays: glutamic acid decarboxylase antibodies (GADA), islet cell antibodies (ICA), and insulin autoantibodies (IAA), routinely. GADA and ICA levels were classified into three groups: (1) the low group included GADA levels of 0–19 U/mL and ICA levels of 1 : 10 titer, (2) the moderate group included GADA levels of 20–199 U/mL and ICA levels of 1 : 32 titer, and (3) the high group included GADA levels of 100 U/mL or higher and ICA levels of 1 : 100 titer. The control group consisted of nonobese and nonoverweight healthy children whose viral IgM antibodies had been studied. We recorded the dates when the blood samples were drawn. One healthy child was chosen for every patient in the same month. If more than five patients were included in the same month, a maximum of five children were chosen for the control group.

The following viruses were studied in every child in the study and control groups: influenza virus A (H1N1, H3N2), with respect to the Köppen-Geiger climate classification for Gaziantep Province [16]. Influenza virus B (IVB); parainfluenza viruses 1, 2, 3, and 4 (PIV1, PIV2, PIV3, and PIV4); adenovirus 3 (AdV3); respiratory syncytial virus (RSV); echovirus 7 (ECHO7), coxsackievirus A7 (CAV7); and coxsackievirus B1 IgM antibodies and coxsackievirus B4 RNA were investigated using PCR. Serum samples were drawn within 1–19 days of admission (mean 6.04 ± 4.32) and stored at −80°C until analysis. Virus IgM antibodies were analyzed with respiratory tract profile 1 kits (Euroimmun, Germany) using the indirect immunofluorescence assay (IFA) test.

We used the composition of the Euroimmun IF kit (FI 2821-1001-1 G “respiratory tract profile 1” IgG field A: verification BIOCHIP). IgM antibodies were studied against the following viruses in the respiratory tract profile kit: RSV, adenovirus type 3, influenza virus type A (H1N1 and H3N2), influenza virus type B, parainfluenza virus types [1–4], echo virus type 7, and coxsackievirus types B1 and A7.

The test procedure was as follows: the fluorescence uptake of the infected cells used as a substrate is observed in all parts of the cell in the case of being positive in pathogen substrates. Positive cytoplasmic fluorescence is seen in viral substrates. The results are reported as “positive” or “negative” according to the fluorescence composition of the infected cells seen in the fluorescence microscope. The dilution ratios that are positive for each viral IgM antibody are given in Table 1. We did not study the IgG antibody against viruses. As this study is limited to viruses, the bacterial IgM antibody results were not given. The RNA extraction process was shown as follows: RNA was extracted using the RNA Purification Kit (Thermo Scientific, Vilnius, Lithuania). RNA templates were mixed in a 50 *μ*L multiplex reaction consisting of 1x buffer, 1.5 mM magnesium chloride (MgCl), 0.2 M deoxynucleotide triphosphate (dNTP) (Thermo Scientific, Lithuania), and 0.4 pmol and 1.25 U Taq polymerase of each primer (Thermo Scientific, Lithuania). The primers and probes of the viral protein (VP1) and VP2 genes of enterovirus were used for enterovirus PCR. As we did not detect any CBV4-specific RNA, RNA sequence analysis was not conducted for CVB4.

This study was approved by the Gaziantep University Clinical Research Ethics Committee (reference number: 387/2017; date: 27 November 2017) for three months. Written informed consent was obtained from the parents/legal guardian of the eligible children before recruitment.

## 3. Statistics

Descriptive statistics, such as mean, standard deviation, and frequency distributions, were used to compare the two groups. A chi-square test was used to compare the presence of viral agents in the patient and control groups. The results were evaluated at a 95% significance level (*p* < 0.05). Statistical analyses were calculated using the Statistical Package for the Social Sciences (SPSS) for Windows version 22.0 pocket program (IBM Corp., Armonk, NY).

## 4. Results

The characteristics of all the participating children at the onset of the newly diagnosed T1DM are shown in Table 2. The study group consisted of 23 boys (57.5%) and 17 girls (42.5%). Six children were Syrian refugees. Seven patients (17%) were younger than three years old. A total of 55% of patients had clinical signs of diabetic ketoacidosis at the onset of T1DM. Two of the patients with newly diagnosed T1DM were classmates who were diagnosed with T1DM in the same month. Both classmates were from the city of Kilis on the border of Gaziantep Province. They were eight years old. The second case presented to our clinic two weeks after the first case. The two children had been admitted with signs of diabetic ketoacidosis in December 2013, and they had also IgM positivity against the same virus profiles (Table 3). Both of the classmates had antiviral IgM antibodies against CAV7, ECHO7, and IVB. In addition, two siblings were admitted to the hospital with diagnosed diabetic ketoacidosis in the same month. Two siblings were admitted with newly diagnosed T1DM in November 2014. One of them was 2 years old, and the other was 10 years old. The boy who was 2 years old was diagnosed with T1DM three weeks after his brother. They had also the same viral IgM antibody (Table 3). The two siblings had antiviral IgM antibodies against IVB, PIV4, and CAV7.

**Figure 1:**
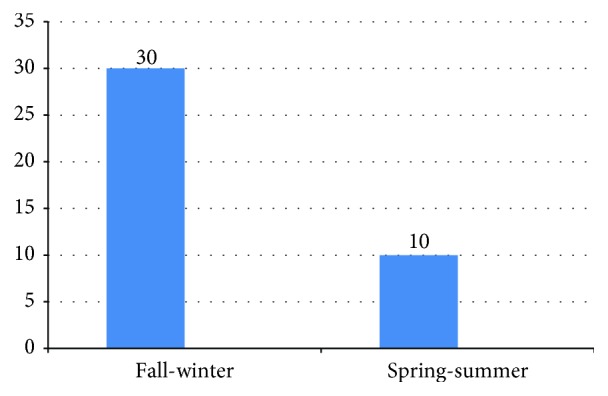
The distribution of patients newly diagnosed with T1DM (*n*) in each season.

**Figure 2:**
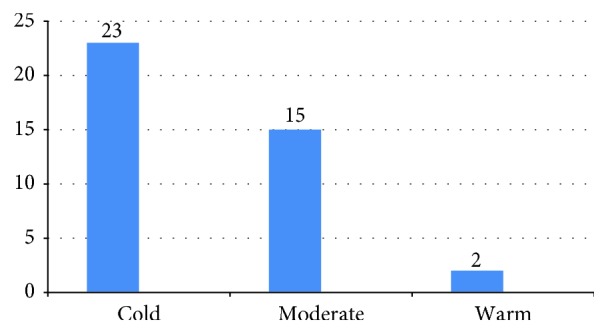
The distribution patients newly diagnosed with T1DM (*n*) in each month group.

The distribution of patients according to season and month is shown in Figures 1 and 2. Thirty patients (75%) were diagnosed in the fall-winter season. The distribution of patients according to month of admission was as follows: October = 7, November = 9, December = 9, January = 3, February = 2, March = 4, April = 4, and May = 2. No patients with newly diagnosed T1DM were admitted in June, July, August, or September. While there was a significant association between high GADA levels and CBV4-seropositive patients (*p* = 0.019), high ICA levels were found, significantly more often in IVB- and ECHO7-seropositive patients (*p* = 0.006 and *p* = 0.007, resp.).

The distribution (*n*) of IgM seropositivity for each virus is shown in Table 3. In terms of comparing the control group with the study group, the most common virus seropositivity and percentages were as follows: IVB (70%), ECHO7 (45%), PIV4 (40%), CAV7 (27.5%), and influenza virus A H3N2 (22.5%). We found that there was a significant association between the IgM seropositivity of these viruses and T1DM, with respect to the control group. Whereas only three patients (10%) had a dual mixed viral IgM antibody in the control group, 24 patients (60%) in the study group had multiple (two or more) viral IgM seropositivity, concurrently.

The incidence of viral IgM seropositivity according to season and month is shown in Figures 1 and 2. In the study group, 75.5% of IgM viral seropositivity was detected in the fall-winter season. In addition to this, 90.1% of viruses were detected in the cold or moderate months. No child in the study group or control group had CBV4 RNA in their serum. In patients with IgM seropositivity of two or more, the mean GADA level (540 ± 718.34) was higher than that in patients with IgM seropositivity of one (mean 63.1 ± 73.39) (*p* = 0.02).

## 5. Discussion

The present study demonstrates that the time of the onset of newly diagnosed T1DM coincided with the fall-winter season, which is the typical season for respiratory tract viruses, and T1DM patients had an association with respiratory viruses and enteroviruses including IVB, ECHO7, PIV4, CAV7, and H3N2 at the clinical onset. These results indicate that enteroviruses and respiratory tract viruses, in addition to seasonal variation, could play a role in the etiopathogenesis and clinical onset of T1DM.

The incidence of T1DM, as well as of other autoimmune diseases, has been steadily increasing around the world. The largest increase has been seen in developed Northern European countries, such as Finland. However, the worldwide increase is occurring too rapidly, which is explained simply by changes in the genetic pool of populations [17]. From an evolutionary perspective, the increasing incidence of T1DM in wealthy countries arises from rapid changes in environmental forces [18]. A strong immune system has developed in human species to ward off many infectious agents. This has simultaneously made it more susceptible to autoimmune diseases in highly hygienic environments [19]. The defective immune response activates the bystander autoreactive cytotoxic T lymphocyte, leading to inhibited immune tolerance. This defective immune reaction skews the T helper 2 response to T helper 1 [20, 21], thus triggering an autoimmune disorder, resulting in islet cell damage. In line with the present data, the cause of the increased incidence of T1DM suggests that it is a trade-off in protection against life-threatening viruses, including respiratory viruses and enteroviruses.

The clinical onset of T1DM exhibits seasonal variations [9]. T1DM usually appears in the fall-winter season, which is seen as the typical season for respiratory infections. Moreover, it has been shown that T1DM incidence increases in the aftermath of outbreaks of influenza, mumps, and rubella. As previously documented in epidemiological reports, our study similarly demonstrated that the majority of newly diagnosed cases of T1DM occurred in the fall-winter season (75%) and during the cold or moderate months (90.1%). In a study involving influenza data from the last 12 years in Turkey, it was reported that influenza viruses A and B repeated every year during the fall-winter season [22]. In the 2013-2014 seasons, while influenza virus A H3N2 (73.6%) and IVB (26.4%) were predominant, H1N1 was detected in only 2% of cases in Turkey [22]. Similarly, in our study, while IVB (70%) and H3N2 (22.5%) were more common, IgM antibodies against H1N1 were not found in the same season.

According to the data obtained from Gaziantep Children's Hospital during 2013-2014, 142 children were newly diagnosed with T1DM. Gaziantep Children's Hospital and Gaziantep University Medical Hospital have a high density of cases with T1DM and are located in the seventh biggest city in Turkey. Gaziantep has a population of 2 million. The study population consisted of patients not only from our own province but also from the surrounding area. This high frequency of patients newly diagnosed with T1DM is due largely to the number of Syrian pediatric patients in our region. According to the Turkish Statistical Institute (TSI), in 2014, the total population of Gaziantep was 1,889,466 and the total population in the 0–14 age bracket was 644,872. The calculated T1DM incidence based on this data was 28.2/105/year for Gaziantep City. In comparison, the incidence of T1DM for Diyarbakir, another large city in the same region, was 7.2/105/year in 2010. Approximately 46.3% of newly diagnosed patients are refugee children. Therefore, we suspect that this is the reason why our province's annual prevalence and incidence of T1DM are significantly higher than those of other provinces [17]. The majority of new T1DM cases diagnosed at Regional Children's Hospital also arose during the fall-winter season. In total, 84.6% of cases were diagnosed in the cold or moderate months during 2013-2014.

Compared with the control group, the IgM positivity to IVB, ECHO7, PIV4, CAV7, and H3N2 viruses in T1DM patients was statistically significant. There have been many studies investigating the relationship between enteroviruses, in particular CVB4 and T1DM [3]. In the present study, CBV4-specific RNA was not found in any blood sample in the patient or control groups. However, the number and type of viruses that cause infections can vary in each region. For example, in a previous study conducted in our country, CBV4 RNA was not detected in either the control or the study group [17].

The sibling of a proband patient and the classmate of another proband patient had the same seropositivity as their probands in the same months, strongly supporting the relationship between T1DM and viral etiology. In a prospective study investigating the intrafamilial spread of enteroviruses, siblings of seropositive patients newly diagnosed with T1DM were also studied at the time of the clinical onset in the proband. The same enterovirus seropositivity was then determined in those same siblings. Subsequently, one sibling was found to have islet antibody positivity, followed by T1DM [15].

It has been reported that multiple viruses, especially enteroviruses, can cause diabetes [23]. Enteroviruses and respiratory viruses growing via replicative cycles lead to progressive and persistent chronic inflammation in target tissues. We serologically detected IgM antibodies against a single virus in 32% (*n* = 23) and mixed viruses in 60% (*n* = 24) of the patients. Each patient had a mean virus seropositivity of 2.34. Correspondingly, it has been reported that mixed viruses can cause fulminant T1DM [4]. Mixed viruses that activate and impact each other can also aggravate the existing damage in target tissue. Likewise, replicative cycles that result in “multiple hits” lead to recurrent and cumulative inflammation in target tissues [1, 11]. When compared with patients with single seropositivity, we found a higher mean GADA in mixed seropositive patients. This finding indicates that multiple viruses may contribute to the exacerbation of damage to tissue. While respiratory viruses and enteroviruses accelerate pancreatic islet disruption by means of multiple hits, on the other hand, they also give rise to the clinical onset of T1DM.

This study is one of the few in Turkey to have investigated the association between T1DM and a large number of enteroviruses and respiratory viruses. At the same time, the diabetogenic effect of viruses on the etiopathogenesis of T1DM is still debated. Therefore, viral evidence indicating the potential for T1DM may be found during seasons when enteroviruses and respiratory viruses are prevalent. When the presence of a causative relationship between T1DM and respiratory viruses is proven, it could provide opportunities for new approaches to predicting, preventing, and treating T1DM.

## 6. Conclusion

In conclusion, this study demonstrated that newly diagnosed T1DM appears during the fall/winter season and during months when the temperature is cold or moderate and coincides with seropositivity for both respiratory viruses and enteroviruses including IVB, ECHO7, PIV4, CAV7, and H3N2 at the time of T1DM diagnosis. These results suggest that respiratory viruses and enteroviruses may play a diabetogenic role in addition to the seasonality of the onset of clinical manifestation of T1DM.

## Figures and Tables

**Table 1:** Serum titer, if positivity is considered for each virus.

Respiratory tract profile 1	Dilution rates (IgM)
Respiratory syncytial virus	1 : 10
Adenovirus type 3	
Influenza virus type A (H1N1)	
Influenza virus type A (H3N2)	
Influenza virus type B	
Parainfluenza type 1	1 : 10
Parainfluenza type 2	
Parainfluenza type 3	
Parainfluenza type 4	1 : 100
Coxsackievirus type B1	1 : 10
Coxsackievirus type A7	
ECHO 7	

**Table 2:** Characteristics of the children in the study and control groups.

	T1DM (*n* = 40)	Control group (*n* = 30)
Onset age^&^ (years), mean ± SD	8.17 ± 3.85	7.50 ± 4.28
Boys/girls (*n*)	23/17	19/11
History of T1DM in the classmate or family of the proband (*n*)	2	
Glucose (mg/dL), mean ± SD	472.63 ± 194.16	87.45 ± 17.34
HbA1c (%)	10.59 ± 2.52	4.7 ± 0.78
Day of admission^∗∗^	6.04 ± 4.32	
C-peptide	0.32 ± 0.21	
Ketoacidosis, *n*/%	22/55	
Thyroid antibodies (*n*) at onset	3 cases	
Celiac antibodies (*n*) at onset	1 case	
Patients (*n*) in year		
2013	15	10
2014	25	20
Patients (*n*) in season		
Fall-winter	30	21
Spring-summer	10	9
Patients (*n*) in months		
Cold	23	19
Moderate	15	10
Warm	2	1
Islet antibodies, *n*/%		
GADA	35/87.5	—
ICA	28/70	—
IAA	19/47.5	—
Number of islet antibodies, *n*/%		
Any	3/7.5	1/3.3
One	7/17.5	
Two	15/37.5	
Three	15/37.5	

^&^At the same time. *n*: number of cases—both classmates and siblings. ^∗∗^Blood samples drawn.

**Table 3:** Distribution of positive antiviral IgM antibodies in the study and control groups.

	Study group (*n* = 40)		Control group (*n* = 30)		*p*
	Positive *n* (%)	Negative *n* (%)	Positive *n* (%)	Negative *n* (%)	
Influenza A					
H1N1	0	40 (100)	0	30 (100)	
H3N2	9 (22.5)	31 (77.5)	1 (3.3)	29 (96.7)	0.023^∗^
Influenza B	28 (70)	12 (30)	8 (26.7)	22 (73.3)	≤0.001^∗^
Parainfluenza					
PIV1	No	40 (100)	No	30 (100)	
PIV2	1 (2.5)	39 (97.5)	1 (3.3)	29 (96.7)	0.836
PIV3	No	40 (100)	No	30 (100)	
PIV4	16 (40)	24 (60)	5 (16.7)	25 (83.3)	0.035^∗^
Coxsackievirus					
CVB1	3 (7.5)	37 (92.5)	No	30 (100)	
CAV7	11 (27.5)	29 (72.5)	No	30 (100)	0.003^∗^
ECHO7	18 (45)	22 (55)	1 (3.3)	29 (96.7)	≤0.001^∗^
AdV3	3 (7.5)	37 (92.5)	No	30 (100)	0.125
RSV	2 (5)	38 (95)	1 (3.3)	39 (96.7)	0.733
Any IgM Ab	—	3 (7.5)	—	20 (66.6)	0.002^∗^
One IgM Ab	13 (32.5)	—	7 (23.3)	—	
Mix IgM Ab					
Two	11 (27.5)	—	3 (10)	—	
Three^∗^	6 (15)	—	No	—	
Four	4 (10)	—	No	—	
Five	3 (7.5)	—	No	—	

PIV: parainfluenza; CVB: coxsackievirus B; CAV7: coxsackievirus A7; AdV3: adenovirus 3. ^∗^Classmates had the same viral IgM antibodies against viruses: CAV7, ECHO7, and IVB, and siblings had the same viral IgM antibodies against viruses: IVB, PIV4, and CAV7.

## Data Availability

'The numerical data analysed during present study used to support the findings of this study are available from the corresponding author upon reasonable request.
